# Interplay between Dysbiosis of Gut Microbiome, Lipid Metabolism, and Tumorigenesis: Can Gut Dysbiosis Stand as a Prognostic Marker in Cancer?

**DOI:** 10.1155/2022/2941248

**Published:** 2022-02-08

**Authors:** Indranil Chattopadhyay, Rohit Gundamaraju, Niraj Kumar Jha, Piyush Kumar Gupta, Abhijit Dey, Chandi C. Mandal, Bridget M. Ford

**Affiliations:** ^1^Dept. of Life Sciences, Central University of Tamil Nadu, Thiruvarur, Tamil Nadu, India; ^2^ER Stress and Mucosal Immunology Lab, School of Health Sciences, University of Tasmania, Launceston, Tasmania, Australia 7248; ^3^Department of Biotechnology, School of Engineering and Technology, Sharda University, Greater Noida 201310, India; ^4^Department of Life Sciences, School of Basic Sciences and Research, Sharda University, Greater Noida 201310, India; ^5^Department of Life Sciences, Presidency University, College Street, Kolkata 700073, India; ^6^Department of Biochemistry, School of Life Sciences, Central University of Rajasthan, Ajmer, Rajasthan, India; ^7^Department of Biology, School of Mathematics, Science and Engineering, University of the Incarnate Word, San Antonio, Texas, USA

## Abstract

The gut bacterial community is involved in the metabolism of bile acids and short-chain fatty acids (SCFAs). Bile acids are involved in the absorption of fat and the regulation of lipid homeostasis through emulsification and are transformed into unconjugated bile acids by the gut microbiota. The gut microbiota is actively involved in the production of bile acid metabolites, such as deoxycholic acid, lithocholic acid, choline, and SCFAs such as acetate, butyrate, and propionate. Metabolites derived from the gut microbiota or modified gut microbiota metabolites contribute significantly to host pathophysiology. Gut bacterial metabolites, such as deoxycholic acid, contribute to the development of hepatocellular carcinoma and colon cancer by factors such as inflammation and oxidative DNA damage. Butyrate, which is derived from gut bacteria such as *Megasphaera*, *Roseburia*, *Faecalibacterium*, and *Clostridium*, is associated with the activation of Treg cell differentiation in the intestine through histone acetylation. Butyrate averts the action of class I histone deacetylases (HDAC), such as HDAC1 and HDAC3, which are responsible for the transcription of genes such as p21/Cip1, and cyclin D3 through hyperacetylation of histones, which orchestrates G1 cell cycle arrest. It is essential to identify the interaction between the gut microbiota and bile acid and SCFA metabolism to understand their role in gastrointestinal carcinogenesis including colon, gastric, and liver cancer. Metagenomic approaches with bioinformatic analyses are used to identify the bacterial species in the metabolism of bile acids and SCFAs. This review provides an overview of the current knowledge of gut microbiota-derived bile acid metabolism in tumor development and whether it can stand as a marker for carcinogenesis. Additionally, this review assesses the evidence of gut microbiota-derived short-chain fatty acids including butyric acid in antitumor activity. Future research is required to identify the beneficial commensal gut bacteria and their metabolites which will be considered to be therapeutic targets in inflammation-mediated gastrointestinal cancers.

## 1. Introduction

Diet and endogenous synthetic pathways are the sources of lipids for normal cells [1]. Circulating lipids are involved in the synthesis of fatty acids, sphingolipids, phospholipids, cholesterol, and isoprenoids in normal cells [2]. Bile acids (BAs), which are stored in the gallbladder, are synthesized as a result of cholesterol catabolism in the liver tissue. BAs are involved in the absorption of fat and the regulation of lipid homeostasis through emulsification [3]. Gut microbiota plays a vital role in the transformation of bile acids into unconjugated bile acids [4]. Dietary pattern contributes significantly to the modulation of gut microbiota, which can serve as a driving force in the development of cancer [5].

Microbes, including archaea, bacteria, bacteriophages, viruses, and fungi, are present in different parts of the human body, such as the oral cavity, lung, gut, skin, breast, and urinogenital systems. Most of the organisms contributing to the microbiome in our body are commensal. The highest microbial diversity is reported in the gastrointestinal tract, particularly in the caecum and proximal colon. The microbial diversity in the gastrointestinal region is influenced by several factors such as the mode of newborn baby delivery, feeding habits of infants, adult food habits and lifestyles, and the genetic factors of the host. Gut microbiota contributes significantly to the immunity and drug metabolism in the host, the digestive capacity of food materials, hormonal regulation in the gut, and neuronal function through the gut brain axis [6]. The gut microbiome enriches the variation of the human genome and provides for substantial strain-level diversity [7]. Lipopolysaccharide (LPS) of Gram-negative bacteria and lipoteichoic acid (LTA) in Gram-positive bacteria are considered virulence factors that modulate the host's innate immune response [8].

Gut microbiota contributes to the conversion of bile acids such as cholic acid (CA) and chenodeoxycholic acid (CDCA) into secondary bile acids through 7*α*-dehydroxylation [9]. Gut microbiota is additionally involved in the production of secondary bile acids such as deoxycholic acid (DCA), lithocholic acid (LCA), choline, and short-chain fatty acids (SCFAs) such as acetate, propionate, and butyrate [4]. Anaerobic bacteria are also crucial in the synthesis of ursodeoxycholate (UDCA) in the colonic region [10]. Acetate, butyrate, and propionate maintain the gut barrier by regulating the tight junction proteins and mucous synthesis [11]. The concentrations of SCFAs are high in the ascending colon (70–140 mM) and become lower in the transverse colon (20–70 mM) and descending colon (20–40 mM) [12]. Firmicutes are requisite for the production of butyrate whereas Bacteroidetes are essential in the production of acetates and propionates. The most abundant SCFA in the colon is acetate [13].

Bile salt hydrolases of gut bacteria such as *Bacteroides fragilis*, *Bacteroides vulgatus*, *Bifidobacterium*, *Clostridium perfringens*, *Listeria monocytogenes*, and *Lactobacillus* are involved in the hydrolysis of conjugated primary bile acids. *Clostridium scindens*, *C. hiranonis*, *C. hylemonae* (*Clostridium cluster XVIa*), and *C. sordellii* (*C. cluster XI*) have enzymes that are key players in the 7*α*/*β*-dehydroxylation pathway [14]. Trimethylamine (TMA) is generated due to metabolism of high-choline and carnitine-containing foods, mainly including fish and red meat by the gut microbiota. Organic cation trimethylamine (TMA), secondary bile acids, deoxycholic acid, lithocholic acid, and modified polyunsaturated fatty acids bind with nuclear receptors such as FXR, PXR, PPAR*α*, and PPAR*γ* cell surface receptors, such as GPR40 and TAAR5 [15]. Gut bacterial metabolite DCA induces the development of gastrointestinal tumors such as hepatocellular carcinoma through inflammation [16]. Bile acids such as DCA, LCA, chenodeoxycholic acid (CDCA), and taurochenodeoxycholic acid (TCDCA) demonstrate carcinogenic activity [17].

Butyrate and propionate have an impact on gut physiology and the immune system, while acetate is a gluconeogenesis and lipogenesis substrate. Firmicutes are mainly involved in the synthesis of butyrate, which has a number of contentious effects in the colon. Although there is a wealth of information on the role of butyrate in cancer prevention, there is no definitive evidence on its role in CRC. Butyrate promotes the growth of normal epithelial cells in the colon. Butyrate and acetate block histone deacetylase, affecting the epigenetic changes that drive CRC formation. Propionate is thought to be less efficient in the inhibition of histone deacetylase as compared to butyrate due to its higher bioavailability and insignificant aggregation in colonocytes. *Faecalibacterium*, *Eubacterium*, *Roseburia*, *Fusobacterium*, *Peptoniphilus*, *Coprococcus*, *Porphyromonas*, *Clostridium*, and *Megasphaera* are butyrate producers in the gut. *Fusobacterium* enhances methylation of the hMLH1 gene and microsatellite instability in CRC [18]. Tumor cells display alteration of lipid metabolism to maintain the demand for energy. Lipid metabolism contributes significantly to tumorigenesis [1]. A higher abundance of DCA induces DNA damage, which enhances the risk of the development of gastrointestinal cancers, such as colon and liver cancer [19]. In this review, we mainly discuss the interaction between the dysbiosis of the gut microbiome and cholesterol/lipid metabolism in the development of cancer.

## 2. Gut Microbiome in Gastrointestinal Cancer

**Figure 1:**
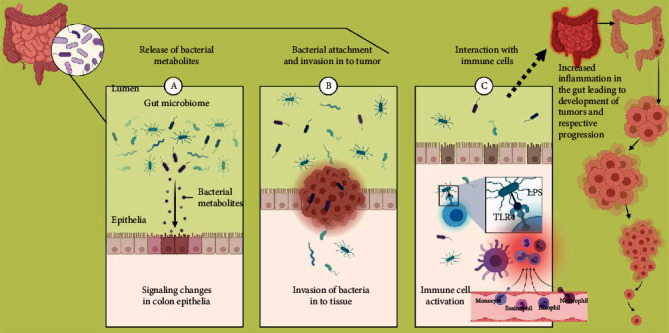
Active role of bacteria and their metabolites in the gut in contributing towards colorectal cancer.

Gut microbiota is enormously involved in the development of gastrointestinal cancers (Figure 1). *Helicobacter pylori*, which colonizes in the gastric epithelium, is responsible for the development of 75% of gastric cancers in the world [20]. This bacterium has several virulence factors such as cytotoxin-associated gene A (CagA), vacuolating cytotoxin (VacA), and outer membrane proteins (OMPs) which are associated with gastric cancer [21]. CagA is decisive in chronic gastritis, mucosa-associated lymphoid tissue lymphoma, and gastric cancer in humans [22]. Colonization of *H. pylori* is responsible for the development of chronic inflammation through overexpression of proinflammatory cytokines such asIL-1*β*, IL-8, IL-17, and TNF-*α*, which enhances the risk of gastric cancer [23]. *Lactobacillus*, *Bifidobacterium*, *Lactococcus*, and *Streptococcus* demonstrate significantly higher abundance in patients with gastric adenocarcinoma [24]. Lactic acid bacteria (LAB) aid in the production of reactive oxygen species (ROS) enhancing DNA damage. LAB contributes to the reduction of nitrate to nitrite which drives mutagenesis, overexpression of protooncogene, enhanced angiogenesis, and inhibition of programmed cell death [24]. Additionally, LAB plays a principal role in epithelial mesenchymal transition [25]. *Escherichia coli* (pks+) succours in the synthesis of colibactin which articulates the development of colorectal carcinoma [26]. *Fusobacterium nucleatum*, *Escherichia coli* NC101, and *Bacteroides fragilis* induce the development of colorectal cancer through activation of the WNT–*β*-catenin signaling pathway [4]. Bacterial genera such as *Bacteroides fragilis*, *Campylobacter*, *Enterococcus*, *Fusobacterium nucleatum*, *Streptococcus*, *Prevotella*, and *Peptostreptococcus* displayed higher abundance, whereas *Bifidobacterium*, *Clostridium* spp., *Lactobacillus*, *Ruminococcus*, and *Roseburia* displayed lower abundance in CRC patients [27].

Bacterial lipopolysaccharides also called microorganism-associated molecular patterns (MAMPs), or pathogen-associated molecular patterns (PAMPs), are recognized by pattern recognition receptors (PPR) such as Toll-like receptors (TLR) on membranes of macrophages and dendritic cells. This receptor transduces the signal through adaptor proteins, such as myeloid differentiation primary response-88 (MyD88) and TIR-domain-containing adapter-inducing interferon-*β* (TRIF), which activate cytokines such as TNF-*α*, IL-1*β*, IL-6, interferon gamma-induced protein10 (IP-10), and interferon-*γ* (IFN-*γ*). The transcriptional factors such as nuclear factor B (NF-kB), activator protein 1 (AP-1), and interferon regulatory factor 3 (IRF3) are key players in this process [28]. This event triggers inflammation which is one of the hallmarks of cancer.

## 3. Hallmark of Lipid Metabolism in Cancer

Phospholipids, fatty acids, triglycerides, sphingolipids, cholesterol, and cholesteryl esters, which are grouped under lipid biomolecules, are the structural components of the plasma membrane and other cellular organelles. These also function as secondary messengers and energy sources [29]. High-density lipoprotein (HDL) and low-density lipoprotein (LDL) demonstrate an association with tumorigenesis [30]. Sterols and isoprenoids, which are the by-product of the mevalonate pathway, contribute to tumor development [31]. Isopentenyl pyrophosphate, farnesyl pyrophosphate, and geranylgeranyl pyrophosphate contribute to inflammation-mediated tumor growth through oncogenic activation of Ras [32]. Low-density lipoprotein receptor (LDLR) showed higher abundance in breast cancer or glioblastoma (GBM) [33]. In cholangiocarcinoma, 22-hydroxycholesterol (22-HC), which is the metabolite of pregnenolone biosynthesis, induces p38-dependent overexpression of the inflammatory protein, cyclooxygenase-2 (COX-2) [34]. 25-Hydroxycholesterol (25-HC) is also involved in the growth of lung, gastric, brain, and breast cancer [35]. 25-HC instigates the growth of glioblastoma via overexpression of the G protein-coupled receptor, 183 [36]. 27-Hydroxycholesterol (27-HC) enhances proliferation and metastasis of ER-positive breast cancer cells through activation of LXR-dependent epithelial-to-mesenchymal transition (EMT) [37]. 27-HC is also crucial in the development of endometrial cancer (EC) and lung adenocarcinoma through activation of STAT3/c-Fos/NFAT [38]. Overexpression of 27-HC activates phosphorylation of AKT and induces secretion of chemokines and cytokines such as IL-6/8, VEGF, MCP-1, and MMPs from CRC cells [39]. 27-HC initiates the development of chemoresistance in prostate cancer through overexpression of androgen receptor and ER*β* [40]. DCA and CDCA induce the development of colon, esophageal, and pancreatic cancer through activation of EGFR, MAPK, NF-*κ*B, and PKC signaling pathways and overexpression of inflammation-inducing proteins, such as COX-2 and prostaglandin E2 (PGE2) [41]. Hyaluronic acid triggers cholesterol efflux from tumor-associated macrophages (TAMs), which escalates the development of tumors through activation of IL-4 and prevention of IFN-*γ* [42]. FA synthesis (FAS) and fatty acid oxidation (FAO) contribute significantly to the development of a tumor [43]. ATP citrate lyase (ACLY), which is involved in the transformation of citrate to oxaloacetate, showed overexpression in gastric adenocarcinoma patients [44]. Overexpression of acetyl-CoA carboxylase2 (ACC2) induces the development of recurrent hepatocellular carcinoma [45]. Overexpression of fatty acid synthase (FASN) has been reported in breast, colon, ovarian, and prostate cancer [15] (Table 1).

## 4. Gut Microbiome in Lipid Metabolism and Its Role in Host Pathophysiology

### 4.1. Bile Acids

Gut bacteria are mainly represented by two predominant phyla, namely, Bacteroidetes and Firmicutes. Other bacterial phyla, such as Actinobacteria, Proteobacteria, Verrucomicrobia, Fusobacteria, and Cyanobacteria, are least abundant in the gut. Bacterial genera, such as *Bacteroides*, *Bifidobacterium*, *Clostridium*, *Eubacterium*, *Escherichia*, *Lactobacillus*, *Peptostreptococcus*, *Propionibacterium*, *Ruminococcus*, and *Streptococcus*, are prevalent in the gut [46]. Bacterial genera *Lactobacillus*, *Streptococcus*, *Staphylococcus*, and *Veillonella* are mainly present in the duodenum and jejunum, whereas *Bacteroides*, *Clostridium*, *Enterobacteria*, *Enterococcus*, *Lactobacillus*, and *Veillonella* are represented in the ileum. These bacteria have a vital role in the deconjugation of bile acids from glycine or taurine through the action of bile salt hydrolases (BSHs) and oxidation of hydroxyl groups [9]. Bacterial bile salt hydrolases (BSHs) are involved in the production of deconjugated BAs and amino acids from conjugated BAs in the gut [47].

The colon constitutes of 10^11-12^ bacteria, which are essentially represented by bacterial phyla Bacteroidetes and Firmicutes. In the colon, bacteria are involved in the transformation of bile acids (CDCA and CA) into secondary bile acids such as LCA and DCA through 7*α*-dehydroxylation [46]. The concentrations of CA modulate the abundance of Firmicutes and Bacteroidetes [48]. Bacterial genera, such as *Bacteroides*, *Bifidobacterium*, *Clostridium*, *Enterococcus*, *Lactobacillus*, and *Listeria*, have bile salt hydrolase activity. BSHs are important in the colonization of pathogenic bacteria such as *Listeria monocytogenes* and *Brucella abortus* in the gut [49]. Hydroxysteroid dehydrogenases (HSDHs), which are predominant in the gut microbiota, belong to bacteria phyla such as Actinobacteria, Firmicutes, and Proteobacteria and are involved in the conversion of bile acid into oxo- (or keto) bile acids through oxidation [13]. Other bacterial enzymes such as hydroxysteroid dehydrogenases (HSDs) are involved in the oxidation/reduction of hydroxy groups of bile acids. Bacterial genera, such as *Clostridium* and *Ruminococcus*, have 3*β*-HSDs, whereas *Bacteroides*, *Clostridia*, *E. coli*, *C. testosteroni*, and *Ruminococcus* spp. have 7*α*- and 7*β*-HSDs. *Acinetobacter* spp., *Brevundimonas* spp., *Cyanothece* spp., *Comamonas* spp., *Fusobacterium* spp., *Nitrosomonas* spp., *Pseudomonas* spp., *Rhodobacter* spp., and *Pseudoalteromonas* spp. have 7*α*-HSD. 12*α*- and 12*β*-HSDs have been identified in *Clostridioides difficile*, *Clostridium leptum*, *Clostridium paraputrificum*, *Clostridium perfringens*, and *Clostridium tertium* [46]. *Methanobrevibacter smithii* and *Methanosphaera stadtmaniae*, which belong to archaea, have BSH which are involved in hydrolyzing both taurine and glycine conjugates [50]. Gut bacterial genera, such as *Bacteroides*, *Bifidobacterium*, *Clostridium*, *Lactobacillus*, and *Listeria*, are crucial in the deconjugation of bile acid, whereas *Bacteroides*, *Clostridium*, *Eubacterium*, *Escherichia*, *Eggerthella*, *Peptostreptococcus*, and *Ruminococcus* are involved in oxidation and epimerization of hydroxyl groups of bile acid. *Clostridium* and *Eubacterium* are involved in 7-dehydroxylation of bile acid. *Bacteroides*, *Eubacterium*, and *Lactobacillus* are involved in the esterification of bile acid. *Clostridium*, *Fusobacterium*, *Peptococcus*, and *Pseudomonas* are involved in desulfation [4]. Anaerobic bacteria genera, such as *Bacteroides*, *Clostridium*, and *Eubacterium*, are involved in the deconjugation of taurine-conjugated and glycine-conjugated bile acids to synthesize unconjugated free forms by using the bile salt hydrolase (BSH) enzyme [9]. Secondary BAs are produced due to the bacterial metabolism of primary BAs, which are generated by the liver [51]. *Bacteroides*, *Clostridium scindens*, *C. hiranonis*, *C. hylemonae*, *C. sordellii*, *Eubacterium*, *Escherichia*, and *Lactobacillu*s are focal in the production of secondary BAs such as DCA and LCA from unconjugated primary bile acids, such as CA, CDCA, and 7*α*-hydroxyl Bas, by using BA-inducible (bai) operon encoding enzymes, such as theCYP7A153 enzyme [9]. *Clostridium perfringens*, *Eubacterium lentum*, and *Ruminococcus gnavus* are involved in the synthesis of iso-bile acids [52] (Table 2).

Metabolites such as bile acids (BAs), lipopolysaccharide (LPS), choline, indole derivatives, and short-chain fatty acids, which are derived from gut microbiota, affect hepatic physiology (Table 3) [53]. The gut microbiota is also engaged in diet-derived choline metabolism, turning it into choline metabolites like trimethylamine (TMA), which is then transformed into trimethylamine oxide (TMAO) in the liver. TMAO induces inflammation in hepatocytes [54]. CA, CDCA, DCA, and LCA are reabsorbed in the intestine and returned to the liver [55]. Hepatocytes synthesize primary bile acids through oxidation of cholesterol by cytochrome P450. Cholesterol hydroxylase enzymes, such as cholesterol 7*α*-hydroxylase (CYP7A1), CYP8B1, and CYP27A133, are involved in the synthesis of primary bile acids, such as cholic acid (CA) and chenodeoxycholic acid (CDCA). CYP7B141 is involved in the production of oxysterol. Bile acid CoA synthetase (BACS) and bile acid-CoA amino acid N-acyltransferase (BAAT) are decisive in the synthesis of taurocholic acid (TCA), taurochenodeoxycholic acid (TCDCA), glycocholic acid (GCA), and glycochenodeoxycholic acid (GCDCA), through the conjugation of CA and CDCA [56]. Unabsorbed bile acids are converted into secondary bile acids, such as deoxycholic acid (DCA) and lithocholic acid, by microbial metabolism [46]. Unconjugated bile acids such as CDCA, LCA, DCA, and CA function as a ligand for several nuclear hormone receptors such as farnesoid X receptor (FXR), pregnane X receptor (PXR), vitamin D3 receptor (VDR), and constitutive androstane receptor (CAR) [57]. Bile acids maintain the growth of commensal microbiota in the gut, integrity of the gut barrier, and host immunity (Table 3) [58]. DCA prevents the growth of *B. fragilis*, *C. perfringens*, *Bifidobacteria*, *Lactococcus*, and *Lactobacillus* and maintains the growth of *Desulfovibrio*, *Dorea*, *Escherichia-Shigella*, and *Ruminococcus* in the gastrointestinal region [59]. Gut microbiota and FXR contribute significantly to bile acid metabolism. Bile acids induce the expression of antimicrobial synthesizing genes, such as Ang1, iNos, and Il18, through FXR [60]. PXR and CAR are actuated by xenobiotics, which are released by the gut microbiota, to activate the overexpression of detoxification enzymes, such as cytochrome P450 [14]. PXR regulates the homeostasis in gut mucosa by altering the gut microbiota. PXR further regulates the infection of *L. monocytogenes* [61]. LCA instigates the activation of VDR, which is involved in the detoxification of toxic bile [62]. Probiotics, such as *Lactobacillus rhamnosus strain* GG and *Lactobacillus plantarum*, aid in overexpression of the VDR gene in intestinal epithelial cells [63]. Fecal microbiota transplantation results in the abundance of *Escherichia*, *Fusobacterium gonidiaformans*, and *Sutterella wadsworthensis* which increases the synthesis of secondary bile acids and short-chain fatty acids [64].

The gut microbiome can influence the metabolism of bile acids and energy homeostasis through activation of farnesoid X receptor and TGR5 [65]. Secondary bile acids, such as DCA and LCA, are critical in maintaining the integrity of intestinal epithelial cells (IECs) by binding to farnesoid X receptor (FXR) [66]. Secondary bile acids trigger anti-inflammatory responses by inhibiting NF-*κ*B activity by binding with G protein-coupled bile acid receptor 1 (GPBAR1) [67]. DCA and LCA initiate cell cycle arrest and programmed cell death through the production of reactive oxygen species (ROS), DNA damage, and overexpression of apoptosis-inducing proteins, such as caspase-3 and poly(ADP-ribose) polymerase (PARP) [68]. Secondary BAs such as DCA and LCA are involved in macrophage-mediated cytokine synthesis in the gastrointestinal tract through interaction with Takeda G protein-coupled BA receptor-5 (TGR5) [69]. TGR5 activates the transformation of the proinflammatory macrophage, M1, to the anti-inflammatory macrophage, M2, which drives the synthesis of IL-10. IL-10 blocks the secretion of proinflammatory cytokines, such as IFN-*γ*, TNF-*α*, and IL-6 [4]. Lithocholic acid (LCA) is a secondary bile acid, which is the metabolic product of chenodeoxycholic acid (CDCA) and ursodeoxycholic acid (UDCA). *Clostridiales* are involved in this transformation by using 7*α*/*β*-hydroxysteroid dehydroxylase enzyme. LCA prevents epithelial-to-mesenchymal transition in breast cancer cells by blocking vascular endothelial growth factor (VEGF) expression [70].

### 4.2. Short-Chain Fatty Acids

Fermentable nondigestible carbohydrate, such as nonstarch polysaccharides, oligosaccharides, lignin, and analogous polysaccharides, are involved in the production of SCFAs, mainly acetate, propionate, and butyrate, through the action of gut microbiota [71]. SCFAs are pivotal in the regulation of the synthesis of gastrointestinal hormones such as ghrelin and leptin from enteroendocrine cells [72]. *Anaerostipes* spp., *Bifidobacterium*, *Coprococcus comes*, *Coprococcus eutactus*, *Coprococcus catus*, *Eubacterium rectale*, *Eubacterium hallii*, *Faecalibacterium prausnitzii*, *Propionibacterium*, *Lactobacillus*, *Roseburia* spp., and *Ruminococcus bromii* are involved in the synthesis of butyrate [73, 74]. *Bacteroides* spp., *Coprococcus catus*, *Dialister* spp., *Salmonella* spp., *Megasphaera elsdenii*, *Roseburia inulinivorans*, *Ruminococcus obeum*, and *Veillonella* spp. are involved in the production of propionate, whereas *Akkermansia muciniphila*, *Bacteroides* spp., *Bifidobacterium* spp., *Blautia hydrogenotrophica*, *Clostridium* spp., *Prevotella* spp., *Ruminococcus* spp., and *Streptococcus* spp. are associated in the synthesis of acetate [75].

**Figure 2:**
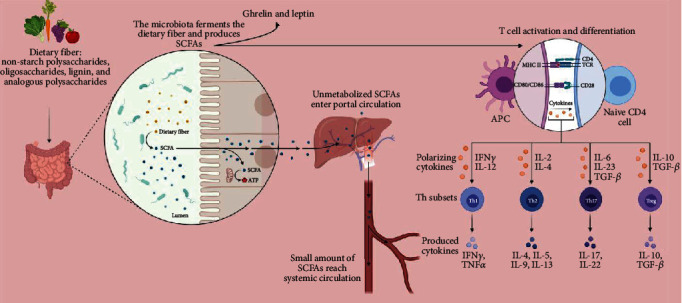
Role of SCFAs in the gut.

SCFAs such as acetate, propionate, and butyrate are produced by gut microbiota and are regarded as prime in the mechanism of lipid metabolism through an interaction between glucagon-like peptide-1 (GLP-1) and G protein-coupled cell surface receptors, such as GPR41 and GPR43 receptors, in the gut [76, 77]. Butyrate, which is derived from gut bacteria such as *Megasphaera*, *Roseburia*, *Faecalibacterium*, and *Clostridium*, is principal in the activation of Treg cell differentiation in the intestine through histone acetylation (Figure 2) [78]. Butyrate prevents the action of class I histone deacetylases (HDAC), such as HDAC1 and HDAC3s which induce the transcription of genes such as p21/Cip1, and cyclin D3, through hyperacetylation of histones driving G1 cell cycle arrest [68]. SCFAs, mainly butyrate, are key in the activation of anti-inflammatory cytokine, IL-10-producing, T cells, through activation of GPR109A-dependent intestinal macrophages [79]. Butyrate triggers the NLRP3 inflammasome via overexpression of GPR43 and GPCR109A in intestinal epithelial cells (IECs), which maintain the integrity of epithelial cells and the gut barrier through inflammatory cytokine (IL-18) secretion [80]. Butyrate acts as a ligand for G protein-coupled receptors (GPR) such as GPR43, GPR41, GPR109A, and Olfr78, which in turn helps in the secretion of the anti-inflammatory cytokine, IL-10, through transformation of CD4+ T cells into immunosuppressive Treg cells [81]. Butyrate prevents proliferation and commences the apoptosis of colon cancer cells through overexpression of p57 and Bax [82]. Additionally, butyrate can impede the proliferation, angiogenesis, and metastasis of colorectal cancer cells through overexpression of sp1, mir-203, and p21/waf-1 and downregulating NRP-1 expression [83]. Acetate is primarily obtained from dairy products, processed meats, and breads. Gut microbiota is involved in the generation of acetate through fermentation of pyruvate. Mitochondrial acetyl-CoA synthetase 1 (ACSS1) and cytoplasmic acetyl-CoA synthetase 2 (ACSS2) utilize acetate as a substrate. Acetyl-CoA synthetase assists in the combination of acetate with CoA to produce acetyl-CoA. Acetyl-CoA carboxylase-*α* aids in the carboxylation of acetyl-CoA, and fatty acid synthase (FASN) on the other hand helps in the condensation of acetyl-CoA and/or malonyl-CoA [84]. Propionate inhibits the growth of hepatocellular carcinoma [85] and cytokine-induced expression of VCAM-1 and ICAM-1 by blocking NF-*κ*B activity which enumerates potent anti-inflammatory potential. *Lactobacillus* and *Bifidobacterium* convert linoleic acid (LA) to conjugated linoleic acids (CLA), which induces programmed cell death through inhibition of PI3K/Akt and ERK signaling pathways [86] (Table 3).

## 5. Role of Bacterial Lipids in Host Pathophysiology

*Bacteroides*, *Porphyromonas*, and *Prevotella* contain sphingolipids. Sphingolipids of *B. fragilis* are engaged in the prevention of invariant natural killer T cell (iNKT) proliferation during neonatal development [87]. Probiotic bacteria, such as *Lactobacillus rhamnosus* GG, are committed in the reduction of *lysophosphatidylcholine*, *sphingomyelin*, and *glycerophosphatidyl choline* levels, whereas *Lactobacillus brevis* and *Streptococcus thermophilus* are involved in the enhancement of neutral sphingomyelinase [88]. Bacteria belonging to the Clostridiaceae/Lachnospiraceae family demonstrate an association with LDL levels, whereas *Eggerthella* demonstrates an association with increased triglyceride levels and *Butyricimonas* show association with reduced levels of triglyceride [89]. Bacterial *β*-glucuronidases, which are encoded by genes such as Gus and BG, are implicated in the deconjugation of conjugated estrogens. *Alistipes*, *Bacteroides*, *Bifidobacterium*, *Citrobacter*, *Collinsella*, *Clostridium*, *Dermabacter*, *Edwardsiella*, *Escherichia*, *Faecalibacterium*, *Lactobacillus*, *Marvinbryantia*, *Propionibacterium*, *Roseburia*, and *Tannerella* have *β*-glucuronidases [90]. Gut commensal bacteria induces the synthesis of norepinephrine and dopamine through the expression of *β*-glucuronidases [91]. Lipoteichoic acid (LTA) is present in Gram-positive bacteria, such as *Bifidobacterium* spp. or *Lactobacilli spp*. LTA induces apoptosis through activation of NO synthase [92]. Whole peptidoglycan (WPG), of the *Lactobacillus paracasei* subsp. *paracasei* M5 strain, inhibits proliferation of HT-29 cells by activating the apoptotic pathway [93].

## 6. Gut Microbiome Modulates Lipid Metabolism in the Development of Cancer

Secondary BAs, such as DCA and LCA, are associated with the production of reactive oxygen and nitrogen species that drive the development of colon cancer through induction of DNA damage [94]. *Clostridium cluster* XIVa aids in the synthesis of secondary bile acids through 7*α*-dehydroxylation of primary bile acids [9]. LCA endorses the growth of colon cancer cells and tumors and activates VDR. Alteration of VDR gene expression leads to the alterations of gut microbiota such as the lower abundance of *Lactobacillus* and higher abundance of *Clostridium* and *Bacteroides*, which propels the progression of CRC [14]. Patients with inflammatory bile diseases (IBD) showed a high risk development of CRC. IBD patients displayed a higher abundance of *E. coli* and a lower abundance of *Faecalibacterium prausnitzii*, which impedes the level of conjugated bile acids and reduces the level of secondary bile acids [95]. Bacterial genera, such as *Clostridium*, *Fusobacterium*, *Peptococcus*, and *Pseudomonas*, which are involved in the desulfation of sulfonated bile acids, demonstrate lower abundance in IBD patients [96]. A lower abundance of butyrate-producing bacteria and secondary bile acid-producing (BSH-rich) bacteria is involved in the development of chronic inflammation in the gastrointestinal tract [95] thereby contributing towards the progression of IBD to CRC [97]. Secondary BAs, such as DCA, initiate proliferation and invasion of colon cancer cells through activation of COX-2, epidermal growth factor receptor (EGFR), extracellular signal-regulated kinases 1 and 2 (ERK1/2), activator protein 1 (AP1), c-Myc, and NF-kB at very low concentration [98]. Cholic acid enhances the abundance of opportunistic gut bacteria such as *Prevotella* and *Desulfovibrio* and reduces the abundance of *Ruminococcus*, *Lactobacillus*, and *Roseburia*, which drive gastrointestinal tumorigenesis through overproduction of the toxic substance, DCA [99].

DCA also induces the development of esophageal cancer [100]. LPS, BAs, and lipoteichoic acid (LTA), which are produced by gut bacteria, induce liver carcinogenesis by suppressing the immune system in the liver [101]. LPS also induces liver carcinogenesis through activation of Toll-like receptor 4 (TLR4) [102]. DCA and LTA are responsible for the development of liver cancer through inducing the synthesis of inflammatory cytokines (IL-6), chemokine (C-X-C motif) ligand (CXCL) 9, and prostaglandin E2 (PGE2) [103]. LTA and DCA induce overexpression of cyclooxygenase-2 (COX-2) and PGE2, which drives immune evasion of tumor cells by suppressing the activity of dendritic cells and natural killer T (NKT) cells; this induces the progression of hepatocellular carcinoma (HCC) [104]. Secondary BAs, such as LCA or omega-muricholic acid (*ω*-MCA), which are produced by *Clostridium*, suppress the activity of sinusoidal endothelial cells in the liver and prevent the aggregation of NKT cells in the liver [105]. *Salmonella enterica* subsp. enterica serovar Typhi and *Salmonella enterica* subsp. enterica serovar Paratyphi demonstrate an association with the development of gallbladder cancer. *S. Typhi* induces the development of secondary bile acids, which drives mutagenic effects in the gallbladder epithelium [106]. BSH-rich bacteria, such as *Enterobacter*, *Enterococcus*, and *Clostridium*, show higher abundance in HCC and are involved in the synthesis of excessive secondary bile acids [55]. Chenodeoxycholic acid (CDCA) induces inflammation of HCT116 colon cancer cells through overexpression of COX-2 [107]. Secondary bile acids, LCA and DCA, bind with GPBAR1, which initiates colorectal cancer by activating EGFR and STAT3 signaling. Interaction between secondary bile acids and GPBAR1 is critical in controlling the activity of proinflammatory macrophages and anti-inflammatory macrophages [108]. CA additionally enhances the growth of opportunistic bacteria, such as *Prevotella* and *Desulfovibrio*, as well as reduces the growth of beneficial bacteria such as *Ruminococcus*, *Lactobacillus*, and *Roseburia*, which enhances DCA synthesis. DCA prevents the growth of *B. fragilis*, *C. perfringens*, *Bifidobacteria*, and *Lactobacilli* and induces the growth of opportunistic bacteria such as *Desulfovibrio*, *Dorea*, *Escherichia-Shigella*, and *Ruminococcus* as well as prevents the growth of beneficial bacteria, such as *Lactobacillus*, *Lactococcus*, and *Roseburia*, which drives the development of gastrointestinal cancer [99].

Activation of FXR in turn induces liver cancer through overexpression of fibroblast growth factor 19 (FGF19) [109]. The expression of FGF19 was significantly elevated in hepatocellular carcinoma patients with a poor prognosis. FGF19 induces proliferation and invasion of hepatocellular carcinoma cell lines [110]. Activated PXR activates proliferation, invasion, and metastasis of colon cancer cells via overexpression of FGF19 [111]. Activated PXR inhibits apoptosis in colon cancer cells (HCT116 and colon LS180) through overexpression of antiapoptotic genes, such as BAG3, BIRC2, and MCL-1, as well as suppression of apoptosis, inducing genes, such as BAK1 and TP53 [112]. High-fat diet (HFD) aids in the generation of secondary bile acids such as DCA and LCA by BSH-rich bacterial genera to include *Enterobacter*, *Enterococcus*, and *Clostridium*, which induce inflammation in the gastrointestinal tract through overexpression of NF-*κ*B and proinflammatory cytokines (TNF-*α* and IL-1*β*). This is common in patients with fatty liver disease, fibrosis, cirrhosis, and hepatocellular carcinoma (HCC). Bile acids help in activating protein kinase C (PKC), which inclines the overexpression of NF-*κ*B through activation of the p38 MAPK pathway. NF-*κ*B induces the activation of inflammation through the secretion of proinflammatory cytokines, such as TNF-*α*, IL-1*β*, and IL-6. IL-6 induces the development of HCC through activation of the JAK–STAT3 pathway, and IL-1*β* enhances the survival of DNA damaged cells in HCC through activation of the phosphoinositide 3-kinase- (PI3K-) MDM2 pathway. A higher abundance of secondary bile acids induces the development of CRC through activation of oxidative damage, overexpression of NF-*κ*B, and inflammation [4] (Table 4). DCA-producing bacteria, such as *Lachnospiraceae* and *Ruminococcaceae*, are responsible for early onset of liver cirrhosis [113]. DCA and CDCA function as immunosuppressive agents. Dysbiosis of BA enhances the translocation of bacteria, which induces infection through disruption of the small intestine barrier. Higher abundance of inflammatory bacteria, such as *Enterobacte*r and *Clostridium*, and a lower abundance of anti-inflammatory bacteria, such as *F. prausnitzii*, *Bifidobacterium*, and *Lactobacillus*, enhance the risk of liver disease. Taurine-conjugated BAs enhance the risk of CRC through the proliferation of sulfate-reducing gut bacteria. Bacteria belonging to the Enterobacteriaceae family demonstrate a positive association with the levels of CDCA and hepatic inflammation [114]. Tumor cells utilize acetate as a carbon source in the synthesis of fatty acids and phospholipids. Overexpression of ACSS2, which is involved in acetate metabolism, has been reported in triple-negative breast cancer, ovarian cancer, glioblastoma, and lung cancer. Overexpression of FASN has been cited in breast cancer. Both ACSS2 and FASN contribute to acetate-dependent lipogenesis in aggressive breast cancer through hypoxia-inducible factor 1*β* (HIF1*β*) [84]. DCA induces metastases of breast cancer cells through FXR [90]. *Bacteroides*, *Prevotella*, and *Porphyromonas* are involved in the synthesis of ceramide phosphoinositol and deoxysphingolipids which are involved in inflammation of the gastrointestinal region.

## 7. Conclusion and Author's Perspective

**Figure 3:**
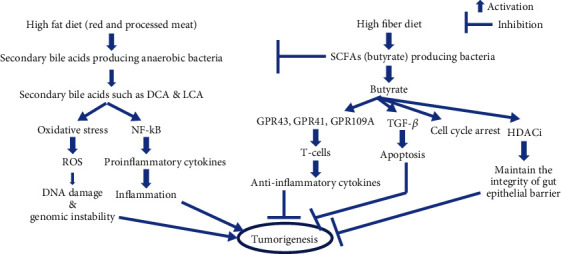
Crosstalk between dietary pattern and gut microbiota-derived lipid metabolism in tumorigenesis.

Gut bacteria-derived or modified metabolites contribute significantly to host physiology. Bile acids are indicated in the metabolism of cholesterol and lipids in our body. Diet and gut bacterial community help in the metabolism of bile acids. Gut microbiota-derived SCFAs demonstrate beneficial effects in host physiology. Bile acid receptors, such as FXR, PXR, CAR, and VDR, are also considered to be therapeutic targets of gastrointestinal cancer. High-fat diets induce tumorigenesis through inflammation and oxidative DNA damage through the actions of gut microbiota-derived secondary bile acids. High-fiber diets prevent the development of tumors through the actions of gut microbiota-derived butyrate (Figure 3). We have discussed the crosstalk between gut microbiota and bile acid metabolism in the development of gastrointestinal cancers, such as CRC and HCC. Drugs targeting bile acid-activated nuclear receptors, such as FXR, PXR, CAR, VDR, and TGR5, might be considered in the treatment of gastrointestinal cancers. Short-chain fatty acids (SCFAs), such as acetate, butyrate, and propionate derived by gut microbiota, demonstrate inhibition of inflammation in gastrointestinal cancer through interaction with G protein-coupled receptors such as GPR41, GPR43, and GPR109A. A higher abundance of secondary bile acids, such as DCA and LCA, in stool and plasma samples resulting from a high-fat diet, may be considered as a diagnostic metabolic biomarker for HCC and CRC patients. These secondary bile acids are involved in the dysbiosis of the gut microbiome. Higher abundance of *Bacteroides*, *Clostridium*, *Desulfovibrio*, *Dorea*, *Enterobacter*, *Enterococcus*, *Escherichia-Shigella*, *Prevotella*, and *Ruminococcus* and a lower abundance of *B. fragilis*, *C. perfringens*, *Bifidobacteria*, *F. prausnitzii*, *Lactobacillus*, *Lactococcus*, and *Roseburia* are considered as diagnostic biomarkers for gastrointestinal cancers.

Metagenomic approaches with bioinformatic analyses are employed to identify the bacterial species in the metabolism of bile acids. It is hence essential to identify the interaction between the gut microbiota and bile acid metabolism to understand bile acid-mediated gastrointestinal carcinogenesis. Metagenomic and metabolomic approaches provide information about the role of metabolites derived from gut bacteria in the development of gastrointestinal cancers. Systems biology approaches are required to understand the liver–bile acid–microbiota axis and its impact on tumorigenesis.

Metabolomic profiling is used for the quantitative measurement of these metabolites in biological samples by using gas chromatography–mass spectrometry (GC-Ms), liquid chromatography–mass spectrometry (LC-MS), and nuclear magnetic resonance (NMR) spectroscopy. These metabolomic approaches are used to study the host-microbiome interaction [116]. Next-generation-sequencing- (NGS-) based metagenomic approaches are used to identify the commensal gut bacteria which are involved in host metabolism and disease progression. Two different sequencing approaches, such as 16S rRNA-based targeted sequencing and shotgun sequencing, are used in the metagenome. Shotgun sequencing approaches are preferred as this approach identifies and characterizes microbial communities. 16S rRNA gene-based sequencing is often limited to taxonomic categorization at the genus level and offers only limited functional characterization. Shotgun metagenomics provides microorganisms to be classified at the species and strain levels. It also provides the functional interactions between hosts physiology and bacterial genomes [4].

Alterations of gut microbiota and the bile acid profile are considered to be therapeutic targets for gastrointestinal cancers. Future research is required to identify the beneficial commensal gut bacteria and their metabolites, which could serve as potential therapeutic targets in inflammation-mediated gastrointestinal cancer.

## Figures and Tables

**Table 1:** Role of lipid metabolites in tumorigenesis.

Cancer	Lipid metabolites	Mode of action in tumor progression	References
Cholangiocarcinoma	22-Hydroxycholesterol (22-HC) metabolite of pregnenolone biosynthesis	Induces p38-dependent overexpression of inflammatory protein cyclooxygenase-2 (COX-2)	[34]
Glioblastoma	25-Hydroxycholesterol (25-HC)	Induces the growth through overexpression of the G protein-coupled receptor 183	[36]
ER-positive breast cancer cells	27-Hydroxycholesterol (27-HC)	Enhances proliferation and metastasis through activation of LXR-dependent epithelial-to-mesenchymal transition (EMT)	[37]
Endometrial cancer (EC) and lung adenocarcinoma	27-HC	Activation of STAT3/c-Fos/NFAT	[38]
Colorectal cancer	27-HC	Activates phosphorylation of AKT and induces secretion of chemokines and cytokines such as IL-6/8, VEGF, MCP-1, and MMPs from CRC cells	[39]
Prostate cancer	27-HC	Induces the development of chemoresistance in prostate cancer through overexpression of androgen receptor and ER*β*	[40]
Colon, esophageal, and pancreatic cancer	DCA and CDCA	Activation of EGFR, MAPK, NF-*κ*B, and PKC signaling pathways and overexpression of inflammation inducing proteins such as COX-2 and prostaglandin E2 (PGE2)	[41]

**Table 2:** List of gut microbiota and its enzymes in bile acid metabolism.

Bacterial genera	Enzymes	Bile acid metabolism	References
*Bacteroides*, *Bifidobacterium*, *Clostridium*, *Enterobacteria*, *Enterococcus*, *Lactobacillus*, *Listeria*, *Streptococcus*, *Staphylococcus*, *Veillonella*	Bile salt hydrolases (BSHs)	Deconjugation of bile acids from glycine or taurine Esterification of bile acid	[4]
*Clostridium*, *Ruminococcus*	3*β*-HSDs	Oxidation/reduction of hydroxy groups of bile acids	[46]
*Bacteroides*, *Clostridia*,*E. coli*,*Eubacterium*, *Peptostreptococcus*,*Comamonas testosteroni*, *Ruminococcus* spp.	7*α*- and 7*β*-HSDs		
*Acinetobacter* spp., *Brevundimonas* spp., *Cyanothece* spp., *Comamonas* spp., *Fusobacterium* spp., *Nitrosomonas* spp., *Pseudomonas* spp., *Rhodobacter* spp., *Pseudoalteromonas* spp.	7*α*-HSD		
*Clostridioides difficile*, *Clostridium leptum*,*Clostridium paraputrificum*, *Clostridium perfringens*, *Clostridium tertium*	12*α*- and 12*β*-HSDs		
*Bacteroides*, *Clostridium*, *Eubacterium*, *Escherichia*, *Lactobacillus*	CYP7A153	Synthesis of secondary bile acids such as lithocholic acid (LCA) and DCA from unconjugated primary bile acids such as CDCA and CA at 7*α*-dehydroxylation	[55]
*Methanobrevibacter smithii* and *Methanosphaera stadtmaniae*	BSH	Hydrolyzing both taurine and glycine conjugates	[50]

**Table 3:** Role of gut bacteria-derived bile acids and SCFA metabolites in host physiology.

Metabolites	Biological function	References
Choline	Lipid metabolism	[54]
Secondary bile acids such as DCA and LCA	Maintaining integrity of intestinal epithelial cells (IECs) by binding with farnesoid X receptor (FXR)	[66]
	Induce anti-inflammatory responses through inhibition of NF-*κ*B activity by binding with G protein-coupled bile acid receptor 1 (GPBAR1)	[67]
	Induce cell cycle arrest and programmed cell death through the production of reactive oxygen species (ROS), DNA damage, and overexpression of apoptosis-inducing proteins such as caspase-3 and poly(ADP-ribose) polymerase (PARP)	[117]
	Involved in macrophage-mediated cytokine synthesis in the gastrointestinal tract through interaction with Takeda G protein-coupled BA receptor-1 (TGR5)	[69]
Lithocholic acid (LCA)	LCA prevents epithelial-to-mesenchymal transition in breast cancer cells by blocking the vascular endothelial growth factor (VEGF) expression	[70]
Butyrate	Activation of differentiation of Treg cell in the intestine through histone acetylation	[78]
	Prevents the action of class I histone deacetylase (HDAC) such as HDAC1 and HDAC3 which induces transcription of genes such as p21/Cip1 and cyclin D3 through hyperacetylation of histone that drives arrest cell at G1 phase	[68]
	Activation of anti-inflammatory cytokine IL-10-producing T cells through activation of GPR109A-dependent intestinal macrophages	[79]
	Induces NLRP3 inflammasome through overexpression of GPR43 and GPCR109A in intestinal epithelial cells (IECs) which maintain integrity of epithelial cell and gut barrier through inflammatory cytokine IL-18 secretion	[80]
	Act as a ligand for G protein-coupled receptors (GPR) such as GPR43, GPR41, GPR109A, and Olfr78 which induces secretion of anti-inflammatory cytokine IL-10 through transformation of CD4+ T cells into immunosuppressive Treg cells	[81]
	Prevents the proliferation and induces the apoptosis of colon cancer cells through overexpression of p57 and Bax	[82]
	It prevents the proliferation, angiogenesis, and metastasis of colorectal cancer through overexpression of sp1, mir-203, and p21/waf-1 and downregulating the expression of NRP-1	[83]
Propionate	Inhibits the growth of hepatocellular carcinoma	[85]
	Prevents the cytokine-induced expression of VCAM-1 and ICAM-1 by blocking the NF-*κ*B activity	[86]
*Lactobacillus* and *Bifidobacterium* are involved in the conversion of linoleic acid (LA) to conjugated linoleic acids (CLA)	Induces programmed cell death through inhibition of PI3K/Akt and ERK signaling pathways	[86]

**Table 4:** Role of gut bacteria-derived bile acid metabolites in tumorigenesis.

Gut bacteria-derived bile acids and SCFAs	Mechanism involved in tumorigenesis	References
LCA	Induces the growth of colon cancer Activates VDR gene expression which induces the alterations of gut microbiota such as the lower abundance of *Lactobacillus* and higher abundance of *Clostridium* and *Bacteroides* which drive the progression of CRC	[14]

DCA	Induces proliferation and invasion of colon cancer cells through activation of COX-2, epidermal growth factor receptor (EGFR), extracellular signal-regulated kinases 1 and 2 (ERK1/2), activator protein 1 (AP1), c-Myc, and NF-kB at very low concentration Induces the growth of opportunistic bacteria such as *Desulfovibrio*, *Dorea*, *Escherichia-Shigella*, and *Ruminococcus* as well as prevented the growth of beneficial bacteria such as *Lactobacillus*, *Lactococcus*, and *Roseburia* which drive the development of gastrointestinal cancer	[98] [99]

Cholic acid	Enhanced the abundance of opportunistic gut bacteria such as *Prevotella* and *Desulfovibrio* whereas reduced the abundance of *Ruminococcus*, *Lactobacillus*, and *Roseburia* which drive gastrointestinal tumorigenesis through overproduction of toxic substance DCA	[99]

DCA and LTA	Responsible for the development of liver cancer through inducing the synthesis of inflammatory cytokines (IL-6), chemokine (C-X-C motif) ligand (CXCL) 9, and prostaglandin E2 (PGE2)	[103]
	Induce overexpression of cyclooxygenase-2 (COX-2) and PGE2 that drive immune evasion of tumor cell by suppressing the activity of dendritic cells and natural killer T (NKT) cells; this induces the progression of hepatocellular carcinoma (HCC)	[104]

Chenodeoxycholic acid (CDCA)	Induces inflammation of HCT116 colon cancer cells through overexpression of COX-2	[107]

LCA and DCA (produced by BSH-rich bacterial genera such as *Enterobacter*, *Enterococcus*, and *Clostridium*)	Bind with GPBAR1 which induces colorectal cancer through activation of EGFR and STAT3 signaling, interaction between secondary bile acids and GPBAR1 is involved in controlling the activity of proinflammatory macrophages and anti-inflammatory macrophages Induce inflammation in the gastrointestinal tract through overexpression of NF-*κ*B and proinflammatory cytokines such as TNF-*α* and IL-1*β* Induce activation of protein kinase C (PKC) which enhances the overexpression of NF-*κ*B through activation of the p38 MAPK pathway; NF-*κ*B induces the activation of inflammation through secretion of proinflammatory cytokines such as TNF-*α*, IL-1*β*, and IL-6; IL-6 induces the development of HCC through activation of the JAK–STAT3 pathway and IL-1*β* enhances the survival of damaged DNA cells in HCC through activation of the phosphoinositide 3-kinase- (PI3K-) MDM2 pathway	[108] [4]

## Abbreviations

SCFAs: Short-chain fatty acids
HDAC: Histone deacetylases
BAs: Bile acids
LPS: Lipopolysaccharide
CDCA: Chenodeoxycholic acid
CA: Cholic acid
DCA: Deoxycholic acid
LCA: Lithocholic acid
UDCA: Ursodeoxycholate
TCDCA: Taurochenodeoxycholic acid
CagA: Cytotoxin-associated gene A
LAB: Lactic acid bacteria
ROS: Reactive oxygen species
LDLR: Low-density lipoprotein receptor
COX-2: Cyclooxygenase-2
27-HC: 27-Hydroxycholesterol
EMT: Epithelial-to-mesenchymal transition
BSHs: Bile salt hydrolases
TMAO: Trimethylamine oxide
BAAT: Bile acid-CoA amino acid N-acyltransferase
FXR: Farnesoid X receptor
PXR: Pregnane X receptor
TGR5: Takeda G protein-coupled BA receptor-5.

## Supplementary Materials

Supplementary Materials Graphical abstract of the article.
