# Supplementary figures and images for: Interplay between Dysbiosis of Gut Microbiome, Lipid Metabolism, and Tumorigenesis: Can Gut Dysbiosis Stand as a Prognostic Marker in Cancer?
Source: Dis Markers. 2022 Feb 8;2022:2941248. doi: 10.1155/2022/2941248 (PMC8847007; doi:10.1155/2022/2941248)

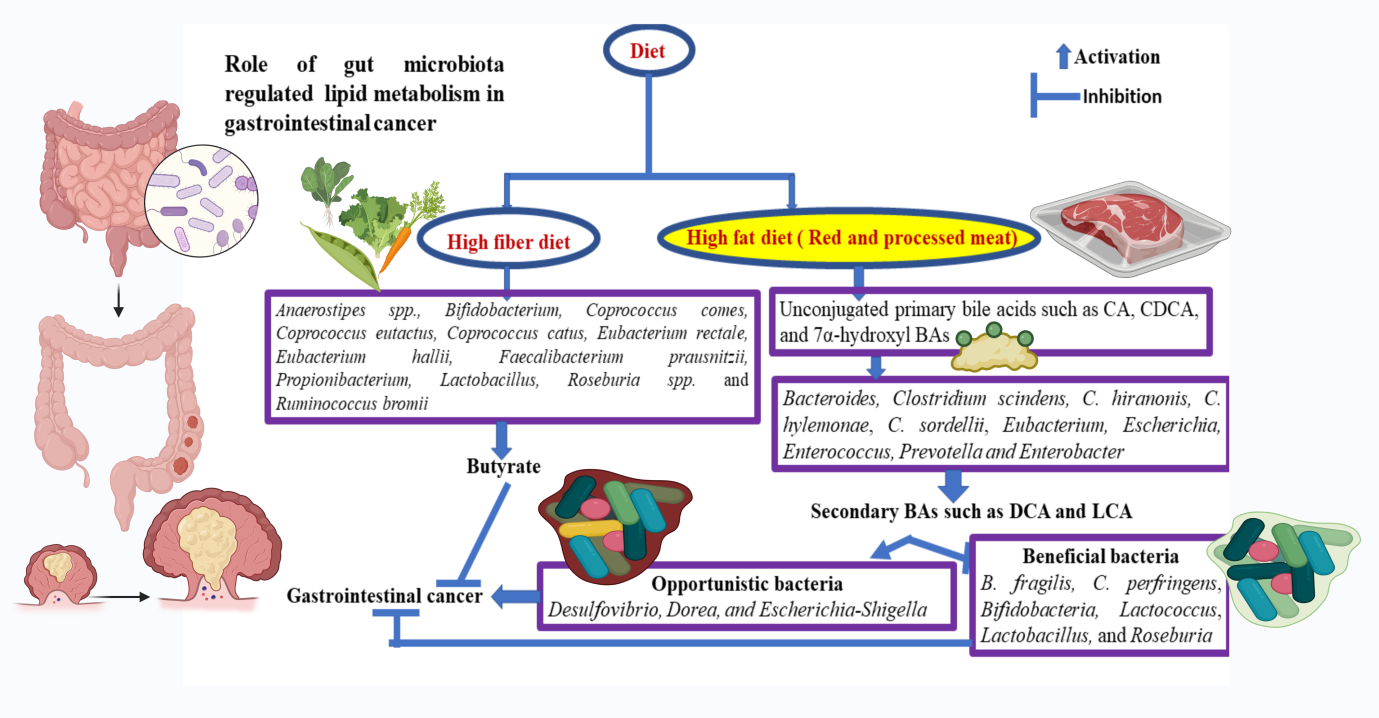


**Graphical Abstract.**

Supplement: Supplementary Materials — Graphical abstract of the article. [file 2941248.f1.zip › Supplementary material_2941248.docx]
